# Financial incentives for smoking cessation in low-income smokers: study protocol for a randomized controlled trial

**DOI:** 10.1186/1745-6215-13-88

**Published:** 2012-06-21

**Authors:** Jean-François Etter

**Affiliations:** 1 Institute of Social and Preventive Medicine, Faculty of Medicine, University of Geneva, CMU – 1 rue Michel-Servet, 1211, Geneva 4, Switzerland

**Keywords:** Financial incentives, Smoking, Smoking cessation

## Abstract

**Background:**

Tobacco smoking is the leading avoidable cause of death in high-income countries. The smoking-related disease burden is borne primarily by the least educated and least affluent groups. Thus, there is a need for effective smoking cessation interventions that reach to, and are effective in this group. Research suggests that modest financial incentives are not very effective in helping smokers quit. What is not known is whether large financial incentives can enhance longer-term (1 year) smoking cessation rates, outside clinical and workplace settings.

**Trial design:**

A randomized, parallel groups, controlled trial.

**Methods:**

*Participants:* Eight hundred low-income smokers in Switzerland (the less affluent third of the population, based on fiscal taxation).

*Intervention:* A smoking cessation program including: (a) financial incentives given during 6 months; and (b) Internet-based counseling. Financial rewards will be offered for biochemically verified smoking abstinence after 1, 2, and 3 weeks and 1, 3, and 6 months, for a maximum of 1,500 CHF (1,250 EUR, 1,500 USD) for those abstinent at all time-points. All participants, including controls, will receive Internet-based, individually-tailored, smoking cessation counseling and self-help booklets, but there will be no in-person or telephone counseling, and participants will not receive medications. The control group will not receive financial incentives.

*Objective:* To increase smoking cessation rates.

*Outcome:* Smoking abstinence after 6 and 18 months, not contradicted by biochemical tests. We will assess relapse after the end of the intervention, to test whether 6-month effects translate into sustained abstinence 12 months after the incentives are withdrawn.

*Randomization:* Will be done using sealed envelopes drawn by participants.

*Blinding:* Is not possible in this context.

**Discussion:**

Smoking prevention policies and interventions have been least effective in the least educated, low-income groups. Combining financial incentives and Internet-based counseling is an innovative approach that, if proven acceptable and effective, could be later implemented on a large scale at a reasonable cost, decrease health disparities, and save many lives.

**Trial registration:**

Current Controlled Trials ISRCTN04019434.

## Background

Tobacco smoking is the leading avoidable cause of death and disease in high-income countries [1]. The smoking-related disease burden is borne disproportionately by the least educated and least affluent people, because of the high smoking prevalence in these groups [2]. To reduce smoking-related health disparities, it is important to design effective smoking cessation programs that reach to low-income smokers. Since this group may be harder to reach with traditional information and education interventions [3,4], other approaches need to be explored. For instance, a strong body of research shows that both participation in addiction treatments and the outcome of these treatments are enhanced by financial incentives [5-8].

### Rationale

Although people have incentives, both financial and health-related, to adopt healthy behaviors, they often fail to do so because these benefits are delayed or intangible. The immediate costs and delayed benefits of behavior change result in decision errors [9]. Giving financial incentives may offset these decision errors and encourage healthy behaviors [5,9]. However, this approach is seldom used, often because of moral considerations (‘people should not be paid to do what they ought to do by themselves’), or due to the assumption that relapse will occur when the rewards are stopped. However, because of the high costs, both material and human, that smoking imposes on society [10], serious consideration must be given to any approach that may effectively and cost-effectively encourage smoking cessation.

In the field of the addictions, interventions using financial incentives are based on a robust theoretical and empirical literature that regards addiction as a form of conditioned behavior, and on well-established behavioral principles of reinforcement [5,7,11]. Research in animals and in humans shows that behavior can be shifted away from drug seeking when drug avoidance is rewarded by alternative reinforcers, if these reinforcers are presented at the right time and are sufficiently rewarding [12]. Furthermore, by promoting abstinence early in the treatment and by improving treatment adherence and retention, financial incentives enable people to take fuller advantage of the other components of clinical treatments [11]. Beside addictions, incentives have been widely used in other fields of medical research, to improve adherence to treatments and to make sure patients present for outcome testing [13].

### Efficacy of incentives for smoking cessation

Two recent meta-analyses concluded that contingency management (i.e. financial incentives) is effective for smoking cessation, producing an effect size of 0.31 to 0.48, which can be considered as a substantial effect [5,7]. A recent review also concluded that financial incentives are efficacious for smoking cessation in economically disadvantaged pregnant women [14]. A third meta-analysis found that financial incentives increased participation in smoking cessation programs, but had no sustained effect on smoking cessation [8]. However, the value of incentives used in most of the studies included in the latter meta-analysis may have been insufficient to produce long-term results (median 120 USD, range 10–750 USD). Furthermore, several of the studies included in these meta-analyses were not designed to produce long-term results. In order to be effective, the value of incentives should be high enough to compete with the reward from smoking and to compensate for withdrawal symptoms (craving, depression, weight gain, and so on) and for the loss of a valued activity. Research showed that in substance abusers, there is a dose–response association between the monetary value of incentives and their effects on abstinence [7]. A recent study showed that financial incentives of up to 750 USD almost tripled smoking cessation rates after 12 months, compared with a no-incentives control group (14.7% *vs.* 5.0%), even in highly educated, affluent workers at a multinational company (65% of them earned > $100,000) [6]. Thus, large incentives are likely to be effective to elicit long-term (12 months) abstinence, even though it is not clear whether these results apply to less educated and less affluent smokers, and outside workplace settings [6].

Almost all of the published studies of financial incentives for smoking cessation were conducted in clinical or worksite settings, and most were relatively short-term and used small incentives [5,7,8,14]. It has not yet been tested whether financial incentives can enhance long-term smoking cessation in a population-based setting, that is, outside clinical and workplace settings. Finally, there is a risk that the effects of incentives may disappear after the incentives are stopped [8,15,16]. Our study will address this important point.

### Incentives: long-term effects

Incentives are external motivators and may not create an enduring commitment to smoking abstinence. Thus, there is a risk that incentives will produce temporary compliance only, and that people will go back to unhealthy behaviors once the incentive program is over [15,16]. This problem is, however, not specific to financial incentives, as the effect of many therapeutic interventions will decline or disappear after the intervention is stopped [17]. Nevertheless, smoking cessation interventions must be designed to produce permanent smoking abstinence, as only permanent abstinence will reduce smoking-related morbidity and mortality. Thus, in addition to initially encouraging quit attempts and participation in smoking cessation programs, the reward schedule should reinforce long-term abstinence, in particular by offering the full amount of rewards only to participants who achieve at least several months of abstinence. Research shows that the relapse rate is relatively small once smokers reach 6 to 12 months of abstinence [17,18]. Thus, we will give the final incentive after 6 months and will conduct the final follow-up after 18 months, to assess relapse 12 months after the end of the incentives program.

### Cost-effectiveness of financial incentives

In the USA, the median cost-effectiveness of 500 medical interventions was 42,000 USD per life year gained [19]. For comparison, the cost-effectiveness of smoking cessation treatments is in the range of 1,400-3,500 USD per life year gained [20,21]. Thus, smoking cessation treatments are about 10 to 20 times more cost-effective than the average medical treatment. In fact, together with vaccines, tobacco dependence treatments are among the most cost-effective of all medical interventions [22]. A review found that the cost-effectiveness of nicotine replacement therapy (NRT) and bupropion is about 1,000 GBP (1,600 CHF) per quitter [20,21]. In our program, financial incentives will cost up to 1,500 CHF per quitter, but previous research shows that on average, participants receive only about 40% to 50% of the maximum reward available [16,23], that is, about 600 to 800 CHF if the maximum reward is 1,500 CHF. This amount is substantially lower than the cost per quitter for pharmacological or medical treatments, considering that over 80% of patients treated in clinical trials fail to quit, and that incentives are paid to successful quitters only. Thus, the planned intervention is likely to be as cost-effective as other smoking cessation interventions, and substantially more cost-effective than the average medical intervention. Finally, previous research shows that financial incentives for patients in addiction treatment are cost-effective [24,25].

## Ethics

### Risks to participants

There is no known risk associated with the Internet-based behavioral support. We think it is unlikely that many non-smokers will smoke before baseline just to test positive on cotinine and CO, in order to be eligible for this study. Besides smoking-related variables, no health-related information will be collected. The files containing the names of participants will be kept confidential and will be accessible only to the first investigator, the computer expert in charge of managing the online data collection system and the research assistant.

### Migros gift cards instead of cash

In order to make sure that the money is not spent on cigarettes, alcohol, or drugs, incentives will be paid exclusively in Migros gift cards. Migros is the largest supermarket chain in Switzerland, and does not sell alcohol or tobacco. It also sells furniture, DIY, garden and sport items, electronic equipment, and has restaurants.

### Informed consent, approval

All participants will sign a consent form (on paper, not online). Participants will receive the intervention at no charge. The study was approved by the ethics committee of the Geneva University Hospitals and is registered in Current Controlled Trials (ISRCTN04019434).

## Methods/design

### Objectives

The objective is to test whether a smoking cessation program combining financial incentives with Internet-based, individually-tailored behavioral support improves smoking cessation rates in low-income smokers, compared with cessation rates observed in a no-incentives control group, and to measure the size of this effect, 12 months after the incentives are stopped, that is, 18 months after the intervention starts. The secondary objectives are: to study whether the outcome is influenced by the characteristics of participants: age, sex and education, tobacco dependence level, motivation to quit (intrinsic/extrinsic [26]), and smoking history; to assess the effect of financial incentives on quit attempts; and to assess whether financial incentives improve use of the online smoking cessation program.

### Design of the trial

A two-arm, open-label, randomized controlled trial with follow-up after 3, 6, and 18 months. We will compare an intervention group that receives financial incentives plus Internet-based support to a control group that receives Internet-based support but no incentives.

#### Randomization

Randomization will take place after we receive the baseline survey and consent form and after we verify eligibility. Randomization will be done with sealed envelopes drawn by participants.

#### Allocation concealment

Participants cannot be blinded to their assignment group. Online data collection will be automatic, and thus, there will be no bias in online assessments. In non-respondents to the online surveys, follow-up data will be collected by postal mail, and in non-respondents to the postal questionnaires, a minimal set of questions (smoking status, quit date, and cigarettes/day) will be asked over the phone.

### Sequence and duration of follow-up

**Figure 1:**
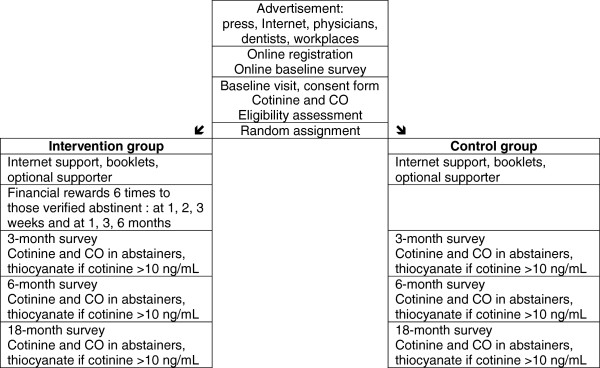
Flowchart of study participants.

Confirmed quitters in the intervention group will receive financial rewards six times: after 1, 2, and 3 weeks, and 1, 3, and 6 months. We will conduct questionnaire surveys and measure saliva cotinine and expired carbon monoxide (CO) levels in both study groups at baseline and after 3 and 6 months (end of the intervention), and after 18 months (that is, 12 months after the end of the intervention, to assess post-intervention relapse) (Figure 1).

#### Timetable and project duration

The preparation phase will last 9 months, the enrolment phase 18 months, the observation phase 18 months, and the data analysis 3 months. Thus, the total duration of the study is 4 years.

### Data

#### Questionnaires

The questionnaires will be collected online, and for non-respondents to the online surveys, by postal mail and over the phone. The questionnaires will be brief and focused, in order to maximize participation. Four waves of questionnaires will be collected in both study groups: at baseline and after 3, 6, and 18 months.

The baseline questionnaire will cover:

Age and sex, school years, occupation, income, body weight and height.

Smoking status, cigarette consumption, level of tobacco dependence.

Motivation to quit (intrinsic/extrinsic) [26], past quit attempts, planned quit date.

Current and past use of smoking cessation treatments and medications.

Address, phone number, e-mail, Internet access.

The follow-up questionnaires (after 3, 6, and 18 months) will cover:

Smoking status (any smoking in the past 7 days, 4 weeks, 6 and 12 months).

Quit date in ex-smokers.

In current smokers: cigarette consumption and level of dependence.

Quit attempts (number, duration, dates).

Motivation to quit (intrinsic/extrinsic), confidence in ability to quit.

Smoking cessation treatments used since enrolment.

Opinions, satisfaction with the intervention.

Items bought with the gift cards by quitters in the intervention group.

Body weight.

Participants will automatically receive an e-mail message inviting them to take part in the online follow-up surveys, 3, 6, and 18 months after their target quit date. Participants who report that they have not smoked any tobacco in the past 7 days will be invited to make an appointment within the next days for biochemical verification.

#### Cotinine, CO, and thiocyanate measurements

Biochemical verification is essential in financial incentives programs. Cotinine and CO tests will be conducted at baseline in all participants (to verify that they are smokers), and at each follow-up (3, 6, and 18 months), in both study groups (intervention and control), only in participants who declare that they have not smoked even a puff of tobacco in the previous 7 days. Self-reported quitters will come to our center to provide CO and saliva. CO tests will be performed with a Bedfont Micro Smokerlyzer, and cotinine in saliva will be assessed with NicAlert tests strips, that can detect cotinine levels >10 ng/mL [27]. Compared with gas chromatography, NicAlert tests strips have a specificity of 95-96%, a sensitivity of 93-99%, a positive predictive value of 95%, and a negative predictive value of 93% [27,28]. In self-reported non-smokers who report using NRT at follow-up and have a negative CO test (0–3 ppm) but a positive cotinine test (NicAlert level > =1, that is, >10 ng/mL) [29,30], we will use thiocyanate to verify smoking abstinence (cutoff: 100 μmol/L) [31,32]. For thiocyanate analysis, saliva samples will be collected in a plastic vial (salivette, Sarstedt) and frozen until sent to the laboratory. Tobacco alkaloids (anabasine or anatabine) are more specific than thiocyanate. However, analyses of anatabine or anabasine are expensive and are not routinely conducted in saliva. In this community setting, taking blood or urine samples will not be feasible.

#### Procedure (see flowchart)

To improve participation in biochemical tests, participants in both study groups will be compensated (Migros gift card, value 25 CHF) for providing saliva and CO samples at follow-up (not at baseline and not when another incentive is given on the same visit). There will be a 6-day time window for the biochemical verification for the first three assessments (that is, after 1, 2, and 3 weeks): people will have to provide the biochemical samples within 6 days of each assessment point. The time window will be 1 month for the 1 and 3 months assessments and 3 months for the 6 and 18 months assessments. These windows were set to maximize participation, because in a community setting, as opposed to workplace or clinical settings, all participants may not strictly keep appointments.

**Figure 2:**
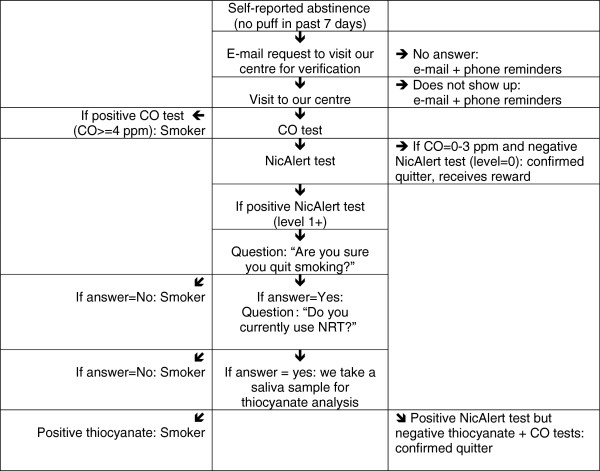
Flowchart for the verification of smoking abstinence.

CO and cotinine tests will be conducted in self-reported quitters only. Those with negative CO tests (0–3 ppm) *and* negative NicAlert tests (level = 0, that is <10 ng/mL) will be declared quitters and will receive their reward immediately, during the same clinic visit. Those who have positive CO tests (> = 4 ppm) will be declared smokers. Those with negative CO tests (0–3 ppm) but positive NicAlert tests (level > =1) will be asked whether they are sure they actually quit. Those who answer ‘yes’ and who currently use NRT will provide a saliva sample for thiocyanate analysis. Those with negative thiocyanate results will be considered quitters (Figure 2). Using CO, cotinine, and thiocyanate allows for a more stringent test of abstinence than in most previous studies of incentives, which used only CO. Carbon monoxide reflects tobacco use during the last few hours, and cotinine and thiocyanate during the last few days only. No biochemical test allows for the verification of abstinence over more than a few days.

### Selection and withdrawal of subjects

#### Enrolment strategies

Participants will be 800 low-income smokers. They will be informed of the study via advertisements in the press, on the Internet, in workplaces, hospitals, pharmacies, clinics and medical and dentistry offices, by direct mailing, and by direct contact with the study assistant in the streets and at sports events. Advertisements will be placed in media and in events that reach to low income, low educated people (for example football and hockey games, free newspapers). The advertisements will inform participants of the possibility of earning up to 1,500 CHF for participating in a smoking cessation study. After answering the online baseline questionnaire, participants will visit our center, where eligibility will be assessed. We define ‘low income’ as a documented taxable annual household income ≤50,000 CHF for single people and < =100,000 CHF for married people, which is the income of roughly the least affluent third of households in Geneva [33].

### Subject inclusion criteria

1. > 18 years old.

2. Taxable income ≤50,000 CHF (single) or < =100,000 CHF (married), proven by most recent fiscal taxation.

3. Smokes at least five cigarettes per day, every day.

4. Has smoked for at least 1 year.

5. Baseline CO reading of at least 10 ppm.

6. Baseline saliva cotinine reading of NicAlert level 1 or higher (> = 10 ng/mL).

7. Sets a quit date within 1 month and commits to quit at that date by signing the quitter contract.

8. Commits to take part in all follow-up surveys and in all biochemical tests.

9. Declares to understand and to accept the control group procedure.

10. Signs informed consent form at each line.

11. Shows identity document with photo (a copy will be kept in our records).

12. Has regular access to Internet and e-mail.

13. Commits to read e-mail daily during the study.

14. Valid e-mail address, postal address and telephone number.

### Subject exclusion criteria

Not meeting all 14 inclusion criteria

In Switzerland, 80% of the general population had Internet access in 2010, including 70% of low-income people and 92% of low-income people younger than 35 years [34]. Thus, requiring regular Internet access may result in a selected subsample of low-income people, but only in those older than 35 years, since in younger people, almost everyone has Internet access.

### The intervention

Tests of financial incentives have almost always been conducted in association with clinical treatment, rather than as stand-alone treatments [7,8]. Thus, in addition to financial rewards, all participants will receive behavioral support. The intervention will have five components:

1 Component for the intervention group only:

1. Financial incentives of up to 1500 CHF, paid to those verified abstinent.

4 Components for both the intervention and the control groups:

2. Internet-based smoking cessation program.

3. Self-help booklets.

4. Optional enrolment of a social supporter of their choice.

5. Quitter contract with quit date (signed by participant, supporter, and study assistant).

Thus, the study will test the effect of financial incentives, over and above a psychosocial intervention comprised of an online program, booklets, a social supporter (optional), and a quitter contract. This is a realistic intervention requiring no medical staff (no physician, nurse, or psychologist). There will be no in-person or telephone counseling, and participants will not receive medication. Thus, if proven effective, this intervention will be implementable at a large scale with reasonable resources.

#### Financial incentives

##### Value of rewards

In substance abusers, greater monetary values of incentives are associated with larger effects on abstinence [7], and small rewards to not produce sustained effects on smoking cessation [8]. Furthermore, the only study that found a sustained (12–18 months) effect of incentives on smoking cessation used large incentives, of up to 750 USD (which corresponded to 950 CHF in 2005 values, when this study was conducted) [6]. In an opinion survey in the US, when asked how much money should be paid to smokers to quit smoking, 53% of participants responded 0 USD, 36% responded 50–500 USD and 11% responded >1,000 USD [35]. When we asked the same question to 120 visitors of the Stop-tabac website, 26% responded 0 CHF, 22% responded 10–500 CHF, and 53% responded >1,000 CHF. Based on this information, we will use incentives of up to 1,500 CHF. However, previous research shows that on average, participants in incentive programs receive only about 40-50% of the maximum reward available [16,23], that is, about 600–800 CHF if the maximal amount available is 1,500 CHF. A reward of 1,500 CHF corresponds to 36% of the monthly income of people who earn 50,000 CHF per year. For reasons stated above, incentives will be paid in Migros gift cards, not in cash.

##### Frequency of rewards

Because relapse occurs rapidly after a quit attempt is initiated, abstinence needs to be reinforced early on [36]. Research shows that incentives have a larger impact if they are delivered at high frequency, soon after the target behavior takes place [11]. Thus, participants will be instructed to present at our center for verification as soon as they achieve 1 week of abstinence, so that abstinence can be reinforced soon after it occurs. Abstinence will be reinforced weekly during the first month, and then after 3 and 6 months.

##### Immediacy of rewards

Research shows that reinforcement works best when the target behavior is followed by the reinforcer without delay [37]. Delayed rewards are discounted, and a smaller amount given now is valued the same as a larger amount given later. In substance abusers, rewards are about twice as effective when they are delivered immediately, during the same clinic visit, than when the payment is delayed [7]. Thus, we will pay rewards immediately after conducting the cotinine and CO tests, during visits at our center. This is why we will use NicAlert tests strips that produce results within a few minutes [27].

##### Escalation and reset

Research shows that escalating systems produce longer periods of smoking abstinence compared with constant rewards [38]. Because we want to reward sustained abstinence rather than initial quit attempts, we will use an escalating scheme and give incentives six times during 6 months (100, 150, 200, 300, 350, and 400 CHF at 1, 2, 3 weeks and at 1, 3, and 6 months, respectively). If people smoke or miss an assessment, the value of the next reward will be reset to the value of the last reward they attained, and the escalation scheme will start again at this value. Incentives will be paid only to people with negative CO and cotinine in saliva, assessed with NicAlert tests strips, and to NRT users who have positive cotinine tests but negative CO tests [27].

### Behavioral intervention for both study groups

#### Quitter contract with quit date

A contract will be signed by each study participant, and countersigned by the research assistant and by the optional social supporter. In this contract, participants will commit to quit at a target quit date set no later than 1 month after enrolment, and to make additional quit attempts at a later date if the first quit attempt fails.

#### Social supporter

Participants who agree will designate a social supporter, preferably a non-smoker, with whom they have a regular, positive association (for example, spouse, friend, or colleague). Social supporters will countersign the participant’s quit contract. Social supporters will sign a consent form, receive a document describing their role in the study, and answer a brief questionnaire to return to us by mail, about their smoking status, age, sex, and address.

#### Internet-based, automatic behavioral support (‘Coach’)

Participants in both study groups will have the opportunity to enrol in an online ‘Coach’, which is an automatic, interactive, smoking cessation program. The ‘Coach’ consists of individually-tailored feedback reports, personal pages with progress graphs and a series of e-mail messages sent automatically over 3 months [3,39]. Participants will also be encouraged to use the other services available on the Stop-tabac.ch website (discussion forums, ‘chats’, testimonials, fact sheets, and so on).

There will be no in-person behavioral support of telephone support. However, participants will be able to call the study assistant for administrative purposes.

#### Booklets

Participants in both the intervention and the control group will receive a series of self-help booklets [3,40]. Finally, we will inform participants if any new, effective treatment of tobacco dependence is discovered during the study.

### Control group procedures

After randomization, participants in the control group will be informed about their group assignment, during the enrolment visit. They will be advised to quit smoking and will be encouraged to use the Stop-tabac website and self-help booklets in the same manner as the intervention group. They will be contacted again only for the follow-up surveys and for the cotinine, CO, and thiocyanate tests after 3, 6, and 18 months. In follow-up surveys, we will ask participants in both study groups whether they obtained smoking cessation support, and the type and amount of support they obtained (Internet, quitline, smoking cessation clinic, physician, medications, and so on). In data analyses, this will enable us to control for the amount of support received by participants.

### Cessation induction *vs.* aid to cessation

Financial incentives may have two types of effects: they may induce quit attempts in people who would not otherwise have tried to quit (cessation induction); and they may increase the success of quit attempts (aid to cessation) [41]. Since all participants will commit to make a quit attempt and set a quit date within 1 month of enrolment, our trial will not be a good test of cessation induction, it will rather be an aid-to-cessation trial. We will nevertheless assess the effect of the intervention on quit attempts, as a secondary outcome.

### Assessment of efficacy

The primary outcome will be continuous smoking abstinence between 6 and 18 months, that is, self-report of no puff of tobacco in the previous 7 days at 6 months plus self-report of no smoking between the 6- and 18-month surveys, not contradicted by CO, cotinine, and thiocyanate measurements. The date for follow-up surveys will be tied to the target quit date set at baseline.

As secondary outcomes, we will also assess:

Biochemically confirmed point prevalence of abstinence after 3, 6 and 18 months.

Abstinence at 3, 6, and 18 months using the ‘Russell Standard’, a recently suggested standard for smoking cessation trials [42].

Quit attempts during the intervention phase (number, duration and dates).

Cigarette consumption, motivation to quit, confidence in ability to quit.

Use of the online smoking cessation program.

### Methods for assessing and recording efficacy parameters

To avoid using paper, questionnaires will be collected online. In non-respondents to the online surveys only, questionnaires will be collected by postal mail and then by phone.

#### Baseline data

After answering the online baseline questionnaire, participants will visit our center in Geneva, where smoking status will be biochemically verified. No smoking cessation counseling will be provided during the enrolment visit.

#### Follow-up after 3, 6, and 18 months

Participants will automatically receive by e-mail a request to answer the online follow-up questionnaires, 3, 6, and 18 months after their target quit date. After six e-mail reminders sent every 3 days, non-respondents to the online surveys will receive the follow-up questionnaires by postal mail. After three reminders by postal mail, non-respondents will be contacted by phone [43]. The telephone survey in non-respondents will cover only a minimal subset of questions (smoking status, quit attempts, quit date and cigarettes/day). After six unsuccessful tries to reach non-respondents by phone at different hours and days of the week, we will contact a next of kin or a friend (whose address was provided at baseline by participants who agree with this procedure) to locate non-respondents. In our previous trials, this procedure enabled us to obtain response rates of about 95% [40,44].

There is a risk that participants in the control group will drop out of the study when they learn about their group allocation, which would represent a threat to the validity of the study. We will make sure we obtain similarly high response rates in both study groups at follow-up.

#### Biochemical verification

The outcome is self-reported prolonged abstinence that is not contradicted by biochemical tests of recent abstinence. After 3, 6, and 18 months, participants who report abstinence (no puff of tobacco in the previous 7 days) will be invited to come to our center to perform a CO test and provide saliva samples for cotinine analysis and, if necessary, thiocyanate analysis. The procedure described above (multiple reminders by e-mail, postal mail, and telephone) will also be used to invite participants for the biochemical tests. After the reminders, a study assistant will offer to smokers who claim abstinence but do not show up for testing to meet them at their home or workplace or at a public space, to collect the CO and saliva samples. The saliva samples will be destroyed after the cotinine and thiocyanate analyses.

### Statistics

The main analysis will be a comparison of the proportions of abstinent smokers in the intervention and control groups. We will use chi-square tests and odds ratios with 95% confidence intervals to compare these proportions. If covariates are needed due to imbalance despite randomization, we will use multivariate logistic regression models instead. We will conduct subgroups analyses, and will use multivariate models to test whether the outcome is influenced by participants’ characteristics. We will assess whether motivation (intrinsic or extrinsic) acts as a mediator in the association between incentives and behavior [26]. Analyses will compare participants who did or did not have a social supporter, or who did or did not use the online Coach, to see if these influence outcome or interact with treatment assignment.

#### Sample size calculation

Based on our previous research in a similar population, we expect a quit rate of about 10% in the control group [39,45]. Previous research showed that financial incentives either have little long-term effect on smoking cessation [8], or an effect size of 0.31 to 0.48 [5,7], but larger incentives have a substantial effect (odds ratio > 3) [6]. A sample size of 800 participants (2 x 400) will enable us to detect a difference between quit rates of 10% in the control group and 17% in the intervention group (odds ratio = 1.84, power = 80%, *P* = 0.05).

#### Intention-to-treat, missing data

For the primary outcome, participants will be evaluated in an intention-to-treat analysis, with all randomized participants in the denominator. Participants with missing data at follow-up will be counted as smokers. We will also conduct sensitivity analyses with different assumptions for missing data. Based on our previous studies, we expect 5% of missing data [46,47].

## Discussion

Combining financial incentives with Internet-based behavioral support is an innovative approach, feasible, easily disseminated, and potentially cost-effective. This study will help us understand whether financial incentives and the Internet can be used to modify health-related behaviors in a population-based setting, and it will provide valuable information on the categories of people in whom this intervention is most effective and on the acceptability of this intervention. This program is of particular interest for low-income populations, in whom smoking prevalence is particularly high. If this study proved that this intervention is effective, this would suggest that, to reduce disparities in smoking due to income and education, tobacco control programs may shift prevention budgets from education and information campaigns to a structured program combining financial incentives with online behavioral support. Therefore, this study has the potential to influence policy decisions. If this program was proven effective, it could be widely disseminated, have a substantial impact on smoking cessation rates, decrease health disparities and save many lives.

### Trial status

The enrolment of participants started in August 2011 and will last until February 2013.

## Abbreviations

CO: Carbon monoxide; DIY: Do it yourself; NRT: Nicotine replacement therapy.

## Competing interests

The authors declare that they have no competing interests.

## Authors’ contributions

J-FE had the idea for the study, wrote the study protocol and this article, obtained funding for the study, and is in charge of the implementation of the trial.

## Author’s information

Jean-François Etter is the author of over 100 publications in peer-reviewed journals, most of them on smoking cessation, and is assistant editor of two of the leading journals in the field: *Addiction* and *Nicotine & Tobacco Research*. He has an extensive experience in conducting randomized trials and in developing and implementing smoking cessation interventions. The planned research is an extension and a development of his previous research.

## Funding

Tobacco Prevention Fund of the Swiss Federal Office of Public Health: 676,000 CHF (560,000 EUR, 690,000 USD).
